# Enhancing Retina Images by Lowpass Filtering Using Binomial Filter

**DOI:** 10.3390/diagnostics14151688

**Published:** 2024-08-05

**Authors:** Mofleh Hannuf AlRowaily, Hamzah Arof, Imanurfatiehah Ibrahim, Haniza Yazid, Wan Amirul Mahyiddin

**Affiliations:** 1 Department of Electrical Engineering, Faculty of Engineering, University of Malaya, Kuala Lumpur 50603, Malaysia; mofleh2@hotmail.com (M.H.A.); imanurfatiehah@gmail.com (I.I.); wanamirul@um.edu.my (W.A.M.); 2 Faculty of Electronic Engineering & Technology, Universiti Malaysia Perlis, Ulu Pauh Campus, Arau 02600, Malaysia; hanizayazid@unimap.edu.my

**Keywords:** retina images, luminosity, contrast, boundary reflection, binomial filter (BF)

## Abstract

This study presents a method to enhance the contrast and luminosity of fundus images with boundary reflection. In this work, 100 retina images taken from online databases are utilized to test the performance of the proposed method. First, the red, green and blue channels are read and stored in separate arrays. Then, the area of the eye also called the region of interest (ROI) is located by thresholding. Next, the ratios of R to G and B to G at every pixel in the ROI are calculated and stored along with copies of the R, G and B channels. Then, the RGB channels are subjected to average filtering using a 3 × 3 mask to smoothen the RGB values of pixels, especially along the border of the ROI. In the background brightness estimation stage, the ROI of the three channels is filtered by binomial filters (BFs). This step creates a background brightness (BB) surface of the eye region by levelling the foreground objects like blood vessels, fundi, optic discs and blood spots, thus allowing the estimation of the background illumination. In the next stage, using the BB, the luminosity of the ROI is equalized so that all pixels will have the same background brightness. This is followed by a contrast adjustment of the ROI using CLAHE. Afterward, details of the adjusted green channel are enhanced using information from the adjusted red and blue channels. In the color correction stage, the intensities of pixels in the red and blue channels are adjusted according to their original ratios to the green channel before the three channels are reunited. The resulting color image resembles the original one in color distribution and tone but shows marked improvement in luminosity and contrast. The effectiveness of the approach is tested on the test images and enhancement is noticeable visually and quantitatively in greyscale and color. On average, this method manages to increase the contrast and luminosity of the images. The proposed method was implemented using MATLAB R2021b on an AMD 5900HS processor and the average execution time was less than 10 s. The performance of the filter is compared to those of two other filters and it shows better results. This technique can be a useful tool for ophthalmologists who perform diagnoses on the eyes of diabetic patients.

## 1. Introduction

Retina images significantly contribute to the diagnosis of various eye-related conditions including diabetic retinopathy [1], macula edema [2], neovascularization [3] and glaucoma [4]. They are not only used for diagnosis but are also instrumental in the monitoring of ocular diseases as changes and progression are observed using these images. Ophthalmologists look for peculiar structures or changes in retina images to identify present or impending problems. Although ophthalmologists can analyze retina images effectively, it is a time-consuming process and when there are many patients, inaccurate diagnosis may occur due to lapses, fatigue or carelessness. Thus, computer-assisted diagnosis of retina images is an indispensable tool that can expedite the process and increase its accuracy [5,6,7,8,9,10]. Retina image quality is important in safeguarding the efficiency of manual or computer-assisted diagnosis. Retina images that have varying illumination, low contrast and haziness may cause incorrect interpretation by ophthalmologists and affect the performance of automated analysis systems [5,6]. 

Images affected by contrast and luminosity unevenness can be corrected by many enhancement approaches proposed by researchers [11,12]. A comprehensive review of all image enhancement techniques is not available, but compilations of well-known enhancement methods in specific areas have been presented by researchers from time to time. Schuch et al. focused on reviewing image enhancement of fingerprint images. Various approaches were reviewed and classified into several categories or models [13]. A review by Saba et al. [14] concentrates on image enhancements of knee joint images where the techniques are divided into frequency and spatial classes. A survey of general enhancement methods by Singh and Mittal also classifies the approaches into frequency and spatial groups [15]. Methods that belong to the spatial domain manipulate the pixels in images by transforming or altering them directly. Many techniques belong to this group inclusive of magnitude and log transforms, alpha rooting and histogram-based operations. Frequency-related techniques perform discrete transformations on entire images using Fourier, cosine, wavelet or other bases. Then, the transformed images are processed and reverted to the original image space [16]. 

In practice, the image enhancement methods are chosen according to the issue to solve, the image model used, the software platform, available toolboxes and the database. Of late, there have been reported works on using neural networks with deep learning for noise removal and image enhancement [15,16,17]. There are also attempts to use metaheuristic optimizations to fine-tune the values of parameters used in established approaches [18,19]. The proposed work can be considered a spatial method where a BF is employed to improve retina images in the RGB domain. Information from all the three channels of the color images is used to enhance the region of interest (ROI). 

Several image enhancement methods for color or grayscale images have been implemented on retinal images. When processing color images in RGB space, many researchers utilize only the green channel [20,21]. Foracchia et al. developed a statistical technique to normalize the background of retina images based on some statistical parameters [22]. Coa et al. implemented a method like the retinex algorithm to enhance retina images. There are four primary procedures comprising padding, contrast improvement, grayscale adjustment and refinement. The background padding is implemented to avoid over-enhancing the retinal boundary. Then all low-frequency components are filtered in the root domain. The visual color of the image changes significantly once the contrast is adjusted. Then, to regain the initial image color, every channel is recalibrated. The contrast is then further enhanced using a refinement step [23]. In earlier works, only the green channel is used to locate abnormalities that are present retinal images [24]. But lately, retina images are processed in color. 

Zhao et al. processed the B, G and R color channels of retina images to enhance them. They found that the luminosity and color of the images were correlated [25]. The G, R and B channels should be changed in the same ratio to preserve color integrity. Rao et al. transformed fundus images into HSV and then L*a*b* space, to modify the corresponding V and L components using gamma and CLAHE, respectively [26]. On the other hand, Mitra et al. converted the images into HIS space before equalizing the histogram of the intensity I. The suggested max–min color correction strategy is used to acquire the final image with suitable color [27]. 

Bala et al. used an adaptive histogram equalization with curvelet and claimed that the method generated better results [28]. Again, some researchers transformed retina images into L*a*b space to enhance the contrast of the L component using CLAHE. They scaled the output range by adopting Hubbard’s retinal image brightness standard [29,30]. Qureshi et al. used a different space called CIECAM02 to obtain its brightness component J. Then, the J value was assigned using non-linear mapping based on threshold. They asserted that the results were better than histogram-based approaches, but in some cases, a greenish color shade was introduced [31]. 

In 2021, Alwazzan et al. utilized the green channel of color images to remove noise from them using the Wiener filter. CLAHE was also needed to improve their contrast and then reunite them with the unaltered blue and red components [32]. Coa et al. presented a new approach by using an intensity transfer strategy. Initially, the image will undergo contrast stretching. Then, an efficient intensity transfer approach is presented that can deliver the necessary illumination for a single channel. This procedure requires a guided image, which could be the original image or another image [33]. Kumar et al. converted fundus into HSV and L*a*b spaces and performed histogram equalization on their intensity components, V and L, respectively. Then, the contrasted results are recombined according to the given weights. The color scheme remained because other components were not touched [34].

Based on the review, there are lots of methods that have been proposed. Retina images can be enhanced either in grayscale or color. Most of the researchers utilized the green channel since the green channel contains more useful information when compared to red and blue channels. Meanwhile, color images also show significant improvement after the enhancement process. Several color spaces have been used to isolate the intensity or luminance component so that traditional enhancement techniques can be applied to it without affecting the color integrity. Sometimes, retina images contain boundary reflection besides the usual contrast and luminosity variability. The bright or hazy reflection occludes the area underneath. In this work, a method to alleviate the problem is presented. The method relies on estimating the background brightness of an image and the subsequent compensation of its non-uniformity. Both the contrast and luminosity of grayscale and RGB color images are enhanced to improve their appearance and statistical parameters. 

The rest of this paper is composed of the following sections. Section 2 briefly reviews the materials and methods of this study. This is followed by the experimental results, and a discussion of the work is provided in Section 4.

## 2. Materials and Methods

**Figure 1:**
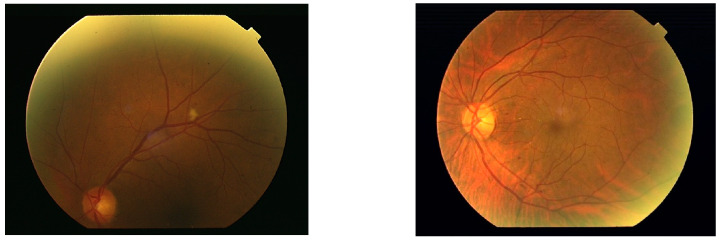
Two samples of retina images with boundary reflection.

In the acquisition stage, the fundus images are usually unevenly illuminated causing contrast and luminosity to vary throughout the ROI. Sometimes they contain boundary reflection as shown in Figure 1. Boundary reflection can obscure abnormalities and other foreground objects like exudates and blood vessels under its locality.

**Figure 2:**
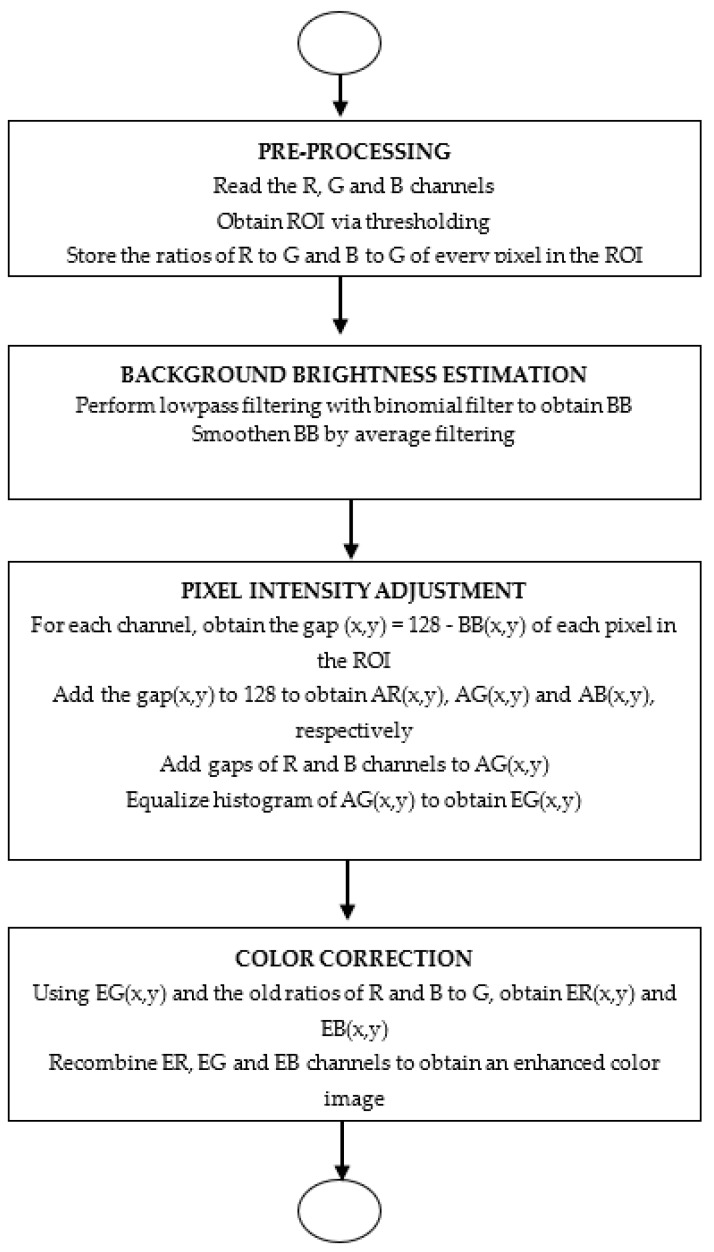
Flow of stages in the enhancement process.

In this work, a method that equalizes the underlying luminosity and enhances the contrast of retina images with boundary reflection is proposed. The main idea is to estimate the background brightness of each pixel in the eye region (ROI) using a binomial filter (BF). This step creates a background brightness (BB) surface for the ROI. Based on the BB, luminosity correction is performed by equalizing the background luminosity of all pixels in the ROI to 128 so that they experience the same brightness. This is followed by contrast adjustment using CLAHE. The approach is implemented in 4 stages involving pre-filtering, background brightness estimation, pixel intensity adjustment, and color correction. Figure 2 shows the flow of stages in the process and the algorithm of the proposed method is presented in Algorithm 1.

First, the red, green and blue (RGB) channels of the retina image are read and stored. Before further processing, the ratios of R to G and B to G at each pixel location in the ROI are computed and stored. These ratios are needed for color correction later. Then, the ROI and its border are localized by thresholding. Once the ROI is available, it can be made symmetrical to the vertical center line and the area outside of the ROI can be set to zero. Often, in an image that is affected by boundary reflection, the reflection center is located along its border. Then, the RGB channels are subjected to average filtering using a 3 × 3 mask to smoothen the RGB values of pixels, especially along the border of the ROI. 

In the background brightness estimation, the ROI of the three channels of an input image is processed one by one. For every pixel in the ROI, a *w* × *w* neighborhood centered at the pixel is established. There are *w*^2^ pixels in the neighborhood (N), including the center pixel. If the center pixel is located near or on the border of the ROI, some of the pixels in the neighborhood (N) will fall outside of the ROI. The pixels in N that lie outside of the ROI are not convolved with the filter coefficients and thus their multiplications are dropped. This step creates the smooth background brightness (BB) surface given by Equation (1).

(1) $$\;\;\;\;\;\;BB\left(x,y\right)=\frac{1}{D}\sum_{n=-w}^{w\;}\sum_{m=-w}^{w}i\left(x-m,\;y-n\right)b(m,n)\;\;\;\;\;$$

where *D* is the sum of all binomial coefficients in *N* that are involved in the convolution

(2) $$\;\;\;\;\;\;D=\sum_{n=-w}^{w\;}\sum_{m=-w}^{w}b(m,n)$$

such that

(3) $$\;\;\;\;\;\sum_{m,n\;\epsilon \;N}^{\;}\frac{b(m,n)}{D}=1\;\;\;$$

Finally, the result is smoothened by average filtering with a 3 × 3 average filter. The output is a slow varying surface of the background brightness *BB*(*x*,*y*). The brightness surfaces of the red, green and blue channels are designated as *BBR*(*x*,*y*), *BBG*(*x*,*y*) and *BBB*(*x*,*y*), respectively. 

The background brightness (BB) approximates the underlying luminosity for each pixel in the ROI. It is a smooth surface that is free of foreground objects. Note that for each pixel, its blue, green and red values, *B*(*x*,*y*), *G*(*x*,*y*) and *R*(*x*,*y*), can be higher or lower than its respective background values provided by the *BBB*(*x*,*y*), *BBG*(*x*,*y*) or *BBR*(*x*,*y*). The difference between the value of a channel and its background brightness is called the channel gap. The adjustment strategy is to level the underlying luminosity of each pixel to the same value of 128 as follows.
**Algorithm 1**: Algorithm of the proposed method**Read** input image **Separate** R, G and B channels **Calculate** ratios of R and B to G **Threshold** the channels to obtain the ROI **Obtain** *BB*(*x*,*y*) of each channel by convolving the ROI with BF **for** each pixel in the ROI of each channel **convolve** with 41 × 41 BF **endSmoothen** the *BB*(*x*,*y*) by 3 × 3 averaging filter **Obtain** the adjusted surface for each channel **for** every pixel *AR*(*x*,*y*) =*128 + R*(*x*,*y*) − *BBR*(*x*,*y*) *AG*(*x*,*y*) =*128 + G*(*x*,*y*) − *BBG*(*x*,*y*) *AB*(*x*,*y*) =*128 + B*(*x*,*y*) − *BBB*(*x*,*y*) **end** **Further improve the green channel** *AGF*(*x*,*y*) =*128 + R*(*x*,*y*) *+ G*(*x*,*y*) *+ B*(*x*,*y*) − *BBR*(*x*,*y*) − *BBG*(*x*,*y*) − *BBB*(*x*,*y*) **Equalize** *AGF*(*x*,*y*) by CLAHE to obtain *EG*(*x*,*y*) **Obtain** the new *R*, *G* and *B* using *EF*(*x*,*y*) and the stored ratios of *R* and *B* to *G*   *Rnew*(*x*,*y*) = *R*/*G* × *EG*(*x*,*y*)   *Bnew*(*x*,*y*) = *B*/*G* × *EG*(*x*,*y*)   *Gnew*(*x*,*y*) = *EG*(*x*,*y*) **end procedure**

For every pixel (*x*,*y*) of the ROI, the background brightness is subtracted from the pixel value to obtain the gap. For instance, for the red channel *R*(*x*,*y*) and its background brightness *BBR*(*x*,*y*), the gap is *R*(*x*,*y*) − *BBR*(*x*,*y*). It can be zero, positive or negative. The adjusted red, *AR*(*x*,*y*), is formed by adding the gap to 128. Then the same adjustment is made to the green and blue channels such that

*AR*(*x*,*y*) = 128 + *R*(*x*,*y*) − *BBR*(*x*,*y*) (4)

*AG*(*x*,*y*) = 128 + *G*(*x*,*y*) − *BBG*(*x*,*y*) (5)

*AB*(*x*,*y*) = 128 + *B*(*x*,*y*) − *BBB*(*x*,*y*) (6)

The last three equations show that at each location (*x*,*y*), the gap is added to the base level of 128. In other words, for each pixel, the gap is maintained although the background brightness is shifted to 128. Throughout the ROI of the adjusted images, the background luminosity appears to be uniformly distributed. After adjusting the background brightness of the ROI to 128, the channels seem to have lost some contrast due to the brighter background. 

The green channel is key to the color correction step of the image in the post processing stage. As such, it should contain the most information since the other two channels will be adjusted based on their ratios to the green channel. From observation, the red channel is more volatile than the green or blue, as most foreground objects contain high values of red. The blue channel is the most stable and it captures the background very well. The green channel usually fluctuates in tandem with the red channel but with less variation. The gaps of the red and blue channels to their respective background brightness surfaces can be added to the green channel to improve its contrast and stability. So, Equation (5) becomes

**Figure 3:**
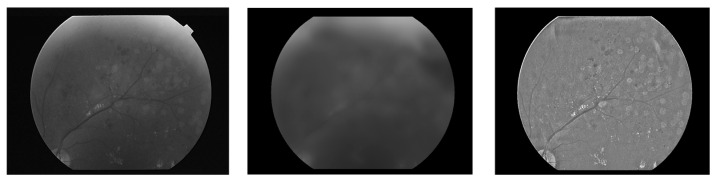
The green channel of an image, its background brightness and fully adjusted form.

*AGF*(*x*,*y*) = 128 + *R*(*x*,*y*) + *G*(*x*,*y*) + *B*(*x*,*y*) − *BBR*(*x*,*y*) − *BBG*(*x*,*y*) − *BBB*(*x*,*y*) (7)

where *AGF*(*x*,*y*) stands for the adjusted green channel with full information. For retina images, this step helps improve the appearance and content of the green channel. Figure 3 shows the green channel *G*(*x*,*y*) of an image, its brightness surface *BBG*(*x*,*y*) and its fully adjusted form *AGF*(*x*,*y*). The size of the BF used is 41 × 41 and its coefficients are scaled so that the highest coefficient is 100 and the lowest is bottom-limited at 1. The 41 × 41 size is chosen because it produces decent results and is not too slow to implement.

Then, the contrast of the *AGF*(*x*,*y*) is further improved by applying contrast-limited adaptive histogram equalization (CLAHE). The operation is implemented on the entire ROI. A constraint is imposed on maintaining the number of grey levels in the image. Due to the constraint, the histogram of the ROI is mainly stretched to improve the contrast of the background and the foreground of the image. The frequency of any value is limited to twice the value of the uniform distribution to restrict the contrast. Another constraint imposed is the gap between consecutive pixel values in the histogram. Each gap can be expanded but should not be reduced to avoid the loss of details. Due to these constraints, the improvement caused by the adaptive equalization to *AGF*(*x*,*y*) is not significant. The outcome of the equalization step is *EG*(*x*,*y*). The red *AR*(*x*,*y*) and blue *AB*(*x*,*y*) can be equalized using CLAHE too.

If the equalized channels are reunited directly, the resulting color image will have peculiar color tones that do not correspond well to the original image. Therefore, the red *AR*(*x*,*y*) and blue *AB*(*x*,*y*) can be disregarded because they are deemed unsuitable for recombination. The new red and blue channels are the stored ratios of B and R over G multiplied by the equalized green channel *EG*(*x*,*y*). As for the new green channel, it is simply *EG*(*x*,*y*) itself since it is the anchor in this color correction. Thus, the new R, B and G channels are given by

*R*_*new*_(*x*,*y*) = *R*/*G* × *EG*(*x*,*y*) (8)

*B*_*new*_(*x*,*y*) = *B*/*G* × *EG*(*x*,*y*) (9)

*G*_*new*_(*x*,*y*) = *EG*(*x*,*y*) (10)

Now, *R_new_*(*x*,*y*), *B_new_*(*x*,*y*) and *G_new_*(*x*,*y*) can be recombined to form a corrected color image.

## 3. Results

The databases which consist of 100 retina images were obtained from the online databases of e-OPHTHA, DIARETDB and EyePACS. About half of the images were selected because they contain strong boundary reflections caused by over exposure. The rest were chosen randomly. Most of the images were resized to make them uniform at approximately 500 × 700. The 1D binomial filter was constructed by convolving vector [1 1] to itself consecutively several times to obtain the desired length (Burt P. (1981)). The way to construct a filter with length 2w + 1 was to perform a cascaded convolution of the [1 1] vector to itself 2w times. After the cascaded convolution, the coefficients were divided by the sum of all coefficients in the filter. The 2D binomial filter was obtained from the vector outer product of a 1D binomial filter to itself. If the 1D binomial filter B(x) is regarded as a column vector with length 2w + 1, taking the cross product of B(x) to its transpose B(x)’ produces a 2D binomial filter B(m,n) whose size is (2w + 1) × (2w + 1).

**Figure 4:**
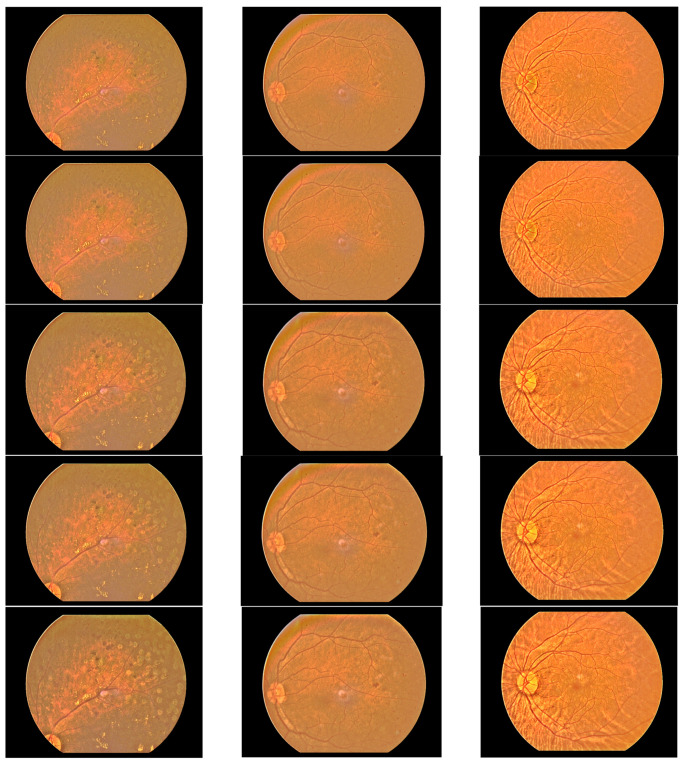
Three retina images processed by BF with α of 1, 0.5, 0.2, 0.1 and 0.01, row wise from top to bottom.

It seemed that the peaky binomial filter was the ideal choice to capture the background brightness of retina images because it was high at the center. Convolving an image with the filter resulted in a *BS*(*x*,*y*) that followed the fluctuation of the data closely but did not capture the underlying trend of the luminosity variation very well. As the size increased, its peakiness became more acute. Hence, the BF coefficients were scaled so that the maximum coefficient is 100 and the minimum is lower-limited at 1. The binomial filter was flattened further by multiplying it with α, where 0.1 < α ≤ 1. Figure 4 shows the samples in Figure 1 that have been processed by a BF whose size is 41 × 41 multiplied with ab α of 1, 0.5, 0.2, 0.1 and 0.01. Take note that when α = 1, the BF is unflattened, and at α = 0.01, all coefficients of the BF become one.

**Figure 5:**
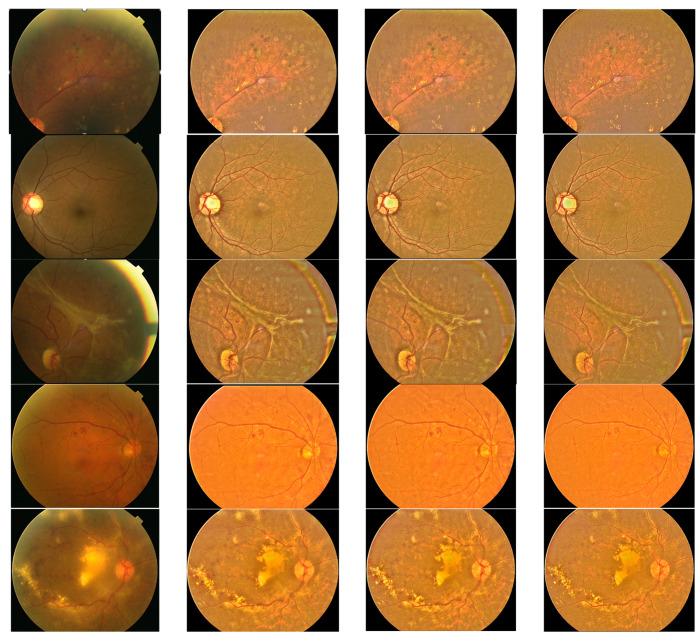

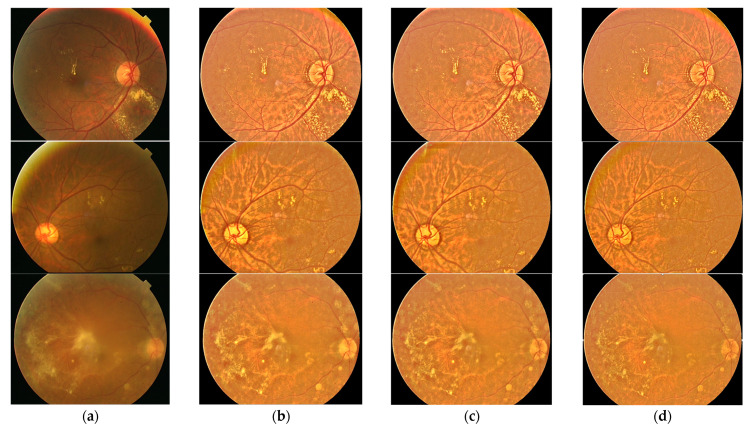
The original, binomial-filtered, median-filtered and Gaussian-filtered samples, column-wise from left to right. (**a**) Original, (**b**) binomial, (**c**) median and (**d**) Gaussian.

It is seen that the output quality improves as α decreases. The 41 × 41 BF with α = 0.1 or 0.01 generates good results for all three samples. The filter with α = 0.1 was chosen for further testing as it was not too flat. Thus, this filter was tested on eight samples and changes in contrast and luminosity were calculated. Its performance was compared to those of median and Gaussian filters. All of the filters had the same size of 41 × 41 and they were executed in the same framework so that the only factor that influenced the results was the filter used. The median filter was implemented using a histogram of pixel values. The Gaussian filter was derived from the standard formula with σ = 5 so that it was not too peaky and then scaled by 10 so that the maximum coefficient value was 10 and the minimum value was limited to 1. Figure 5 shows the results produced by the three filters.

The results look similar as the filters were implemented in the same framework. All images show enhancement in luminosity and contrast throughout the ROI, even in the areas that are partially occluded by reflection. The improvement can be verified visually and quantitatively. The luminosity of the images was obtained by converting them from RGB into L*a*b space, where L stands for luminosity. The average luminosity gain of an image was calculated using equation 11 to estimate the improvement introduced by the proposed method.

(11) $$Luminosity\;Gain=\frac{Avg\;Filtered\;Luminance-Avg\;Unfiltered\;Luminance}{Avg\;Unfiltered\;Luminance}\times 100\%$$

The contrast was estimated using the metric of Matkovic et al. (2005). The local contrasts were the averages of absolute differences of one pixel and its eight nearest neighbors at different resolutions. The global contrast factor (GCF) for the ROI was the aggregate of all local contrasts multiplied by defined weightings. In the experiments, three local contrasts were calculated at three resolutions, and they were multiplied by three weightings of 0.12, 0.142 and 0.154, respectively. These weightings were suggested by the authors themselves. The GCF, or contrast, was calculated before and after filtering. The contrast gain for the images was given by Equation (12) below.

(12) $$Contrast\;Gain=\frac{Avg\;Filtered\;Contrast-Avg\;Unfiltered\;Contrast}{Avg\;Unfiltered\;Contrast}\times 100\%\;\;$$

The contrast and luminosity gains for the eight samples and their averages are presented in Table 1. The figures show that on average, contrast and luminosity gains for the samples are nearly identical for the three filters. Tests carried out on the remaining 92 images revealed similar outcomes with nearly the same figures. Comparatively, the BF performed slightly better than the other two filters. Therefore, it is fair to conclude that the contrast and luminosity of the images have been enhanced by the framework.

## 4. Discussion and Conclusions

It is observed that the test images in Figure 5 contain various levels of contrast and illumination variations. Four of them appear to have boundary reflections of various degrees. After processing, marked improvements in contrast and luminosity are observed in all images. The method manages to remove the boundary reflection and expose the surface with some foreground objects. The second and last samples suffer from a dim appearance, but their corrected versions are brighter. The third image has an intense boundary reflection that splits into two parts. The bottom part is small enough that it might be mistaken as a foreground object. In its corrected form, the boundary reflection is removed but traces around its edge remain. This is because there exists a strong transition of red and yellow around its edge which looks like rainbow stripes. The method considers them as foreground objects and decides to keep them. The same observation applies to sample 6, where a red stripe remains near its top border.

The performance of the method was further tested on the remaining 92 test images of the database and the average gains in contrast and luminosity for the binomial filter were above 170% and 60%, respectively. The median and Gaussian filters achieved slightly less gain for contrast and about the same gain for luminosity. After recombination, the resulting color images resembled the inputs in color tones and distribution, but they showed marked improvement in contrast and luminosity. The framework was executed on MATLAB R2021b powered by an AMD 5900HS processor. The average execution times for the three filters were less than 10 s.

The method works best when the boundary reflection varies smoothly. If the reflection has a steep transition at its border, it is regarded as a foreground object that needs to be preserved. On the contrary, if a large exudate happens to be located at the boundary of the ROI and if its border has a smooth transition, it can be misconstrued as boundary reflection. In this case, it might be eliminated.

In summary, compared to the original images, marked improvements in contrast and luminosity are observed in all processed images. In the boundary areas that are previously shaded by the reflection, the method manages to expose the surface underneath. Overall, the processed images look better than their raw counterparts. The luminosity of the filtered images looks uniform, and the original color shades of the ROI are well preserved. All the foreground objects, including exudates, blood spots, optic discs and blood vessels, are clearly contrasted.

## Figures and Tables

**Table 1:** The luminance (L) and contrast (C) of the samples before and after filtering.

Sample	Before Filtering		Binomial Filtered		Median Filtered		Gaussian Filtered	
	L	C	L	C	L	C	L	C
1	33.0	0.37	57.6	1.05	57.4	1.05	57.5	1.04
2	32.3	0.48	56.8	1.40	56.6	1.27	56.7	1.35
3	30.5	0.38	57.3	1.21	57.7	1.08	56.9	1.18
4	37.15	0.39	64.1	1.13	64.3	1.12	64.2	1.09
5	43.0	0.50	59.43	1.21	60.0	1.23	59.0	1.20
6	35.39	0.62	60.7	1.69	60.5	1.58	60.3	1.66
7	33.48	0.38	60.2	1.11	60.22	1.03	60.4	1.05
8	42.57	0.42	59.7	1.22	59.6	1.10	59.6	1.13
Average	35.92	0.44	59.44	1.25	59.3	1.18	59.3	1.21
Gain	-	-	65.4%	184.6%	65.3%	168.2%	65.2%	175%

## Data Availability

Publicly available datasets were analyzed in this study. This data can be found here: [https://www.adcis.net/en/third-party/e-ophtha/, accessed on 18 March 2024], [https://www.kaggle.com/datasets/nguyenhung1903/diaretdb1-standard-diabetic-retinopathy-database, accessed on 18 March 2024], [https://www.kaggle.com/datasets/deathtrooper/glaucoma-dataset-eyepacs-airogs-light-v2/data, accessed on 18 March 2024].

## References

[1] Sebastian A., Elharrouss O., Al-Maadeed S., Almaadeed N. (2023). A survey on diabetic retinopathy lesion detection and segmentation. Applied Sciences..

[2] Mathews M.R., Anzar S.M. (2021). A comprehensive review on automated systems for severity grading of diabetic retinopathy and macular edema. Int. J. Imaging Syst. Technol..

[3] Tang M.C.S., Teoh S.S., Ibrahim H., Embong Z. (2021). Neovascularization detection and localization in fundus images using deep learning. Sensors.

[4] Sarhan A., Rokne J., Alhajj R. (2019). Glaucoma detection using image processing techniques: A literature review. Comput. Med. Imaging Graph..

[5] Xiao D., Bhuiyan A., Frost S., Vignarajan J., Tay-Kearney M.L., Kanagasingam Y. (2019). Major automatic diabetic retinopathy screening systems and related core algorithms: A review. Mach. Vis. Appl..

[6] Vives-Boix V., Ruiz-Fernández D. (2021). Diabetic retinopathy detection through convolutional neural networks with synaptic metaplasticity. Comput. Methods Programs Biomed..

[7] Tavakoli M., Jazani S., Nazar M. (2020). Automated detection of microaneurysms in color fundus images using deep learning with different preprocessing approaches. Medical Imaging 2020: Imaging Informatics for Healthcare, Research, and Applications.

[8] Kang Y., Fang Y., Lai X. (2020). Automatic detection of diabetic retinopathy with statistical method and Bayesian classifier. J. Med. Imaging Health Inform..

[9] Das S., Saha S.K. (2022). Diabetic retinopathy detection and classification using CNN tuned by genetic algorithm. Multimed. Tools Appl..

[10] Chudzik P., Majumdar S., Calivá F., Al-Diri B., Hunter A. (2018). Microaneurysm detection using fully convolutional neural networks. Comput. Methods Programs Biomed..

[11] Palanisamy G., Ponnusamy P., Gopi V.P. (2019). An improved luminosity and contrast enhancement framework for feature preservation in color fundus images. Signal Image Video Process..

[12] Gupta B., Tiwari M. (2019). Color retinal image enhancement using luminosity and quantile-based contrast enhancement. Multidimens. Syst. Signal Process..

[13] Schuch P., Schulz S., Busch C. (2018). Survey on the impact of fingerprint image enhancement. IET Biom..

[14] Saba T., Rehman A., Mehmood Z., Kolivand H., Sharif M. (2018). Image enhancement and segmentation techniques for detection of knee joint diseases: A survey. Curr. Med. Imaging.

[15] Singh G., Mittal A. (2014). Various image enhancement techniques—A critical review. Int. J. Innov. Sci. Res..

[16] Qi Y., Yang Z., Sun W., Lou M., Lian J., Zhao W., Deng X., Ma Y. (2021). A comprehensive overview of image enhancement techniques. Arch. Comput. Methods Eng..

[17] Soundrapandiyan R., Satapathy S.C., PVSSR C.M., Nhu N.G. (2021). A comprehensive survey on image enhancement techniques with special emphasis on infrared images. Multimed. Tools Appl..

[18] Vijayalakshmi D., Nath M.K., Acharya O.P. (2020). A comprehensive survey on image contrast enhancement techniques in spatial domain. Sens. Imaging.

[19] Sahu S., Singh A.K., Ghrera S.P., Elhoseny M. (2019). An approach for de-noising and contrast enhancement of retinal fundus image using CLAHE. Opt. Laser Technol..

[20] Mazlan N., Yazid H., Arof H., Mohd Isa H. (2020). Automated microaneurysms detection and classification using multilevel thresholding and multilayer perceptron. Journal of Medical and Biological Engineering..

[21] Cao L., Li H., Zhang Y. (2020). Retinal image enhancement using low-pass filtering and α-rooting. Signal Process..

[22] Foracchia M., Grisan E., Ruggeri A. (2005). Luminosity and Contrast Normalization in Retinal Images. Med. Image Anal..

[23] Yang L., Yan S., Xie Y. (2021). Detection of microaneurysms and hemorrhages based on improved Hessian matrix. Int. J. Comput. Assist. Radiol. Surg..

[24] Mayya V., Kamath S., Kulkarni U. (2021). Automated microaneurysms detection for early diagnosis of diabetic retinopathy: A Comprehensive review. Comput. Methods Programs Biomed. Update.

[25] Zhou M., Jin K., Wang S., Ye J., Qian D. (2017). Color retinal image enhancement based on luminosity and contrast adjustment. IEEE Trans. Biomed. Eng..

[26] Rao K., Bansal M., Kaur G. (2022). A hybrid method for improving the luminosity and contrast of color retinal images using the JND model and multiple layers of CLAHE. Signal Image Video Process..

[27] Mitra A., Roy S., Roy S., Setua S.K. (2018). Enhancement and restoration of non-uniform illuminated fundus image of retina obtained through thin layer of cataract. Comput. Methods Programs Biomed..

[28] Anilet Bala A., Aruna Priya P., Maik V. (2021). Retinal image enhancement using adaptive histogram equalization tuned with nonsimilar grouping curvelet. Int. J. Imaging Syst. Technol..

[29] Dissopa J., Kansomkeat S., Intajag S. (2021). Enhance Contrast and Balance Color of Retinal Image. Symmetry.

[30] Vonghirandecha P., Karnjanadecha M., Intajag S. (2019). Contrast and color balance enhancement for non-uniform illumination retinal images. Teh. Glas..

[31] Qureshi I., Ma J., Shaheed K. (2019). A hybrid proposed fundus image enhancement framework for diabetic retinopathy. Algorithms.

[32] Alwazzan M.J., Ismael M.A., Ahmed A.N. (2021). A hybrid algorithm to enhance colour retinal fundus images using a Wiener filter and CLAHE. J. Digit. Imaging.

[33] Cao L., Li H. (2020). Enhancement of blurry retinal image based on non-uniform contrast stretching and intensity transfer. Med. Biol. Eng. Comput..

[34] Kumar R., Bhandari A.K. (2022). Luminosity and contrast enhancement of retinal vessel images using the weighted average histogram. Biomed. Signal Process. Control.

